# The impact of English usage on social media on college students’ interpersonal communication intentions: a dual mediating model of language confidence and English cultural identity

**DOI:** 10.3389/fpsyg.2025.1692474

**Published:** 2025-12-17

**Authors:** Yichao Zhang, Yiming Wu, Yanjun Chen, Hongtao Zhang

**Affiliations:** 1 Zhejiang Province Key Think Tank: Institute of Ecological Civilization, Zhejiang A&F University, Hangzhou, China; 2 Rural Revitalization Academy of Zhejiang Province, Zhejiang A&F University, Hangzhou, China

**Keywords:** social media English usage, interpersonal communication intentions, language confidence, English cultural identity, mediating model

## Abstract

**Introduction:**

Social media has become part of daily life in the era of globalization and the swift development of internet technology. The worldwide user base is expected to go over 4 billion by 2024, radically transforming social interaction patterns. Being active users of social networks, college students frequently use English on these platforms, and one can expect that their readiness to communicate with others can be affected by such use. Available studies have, however, not explored this relationship holistically. This study examines how the use of English in social media influences college students’ willingness to engage in interpersonal communication, with emphasis on the dual and chained mediation relationships between language confidence and English cultural identity.

**Methods:**

The participants completed an online survey via Questionnaire Star using convenience sampling (*n* = 412), targeting college students who use English on social media across different universities in the country. The revised Social Media English Usage Scale, Interpersonal Interaction Intentions Scale, Language Confidence Scale, and Cultural Identity items operationalized as English cultural identity were used to gather data. Correlation analyses and descriptive statistics were conducted in SPSS 26.0, and the chained mediation model was tested with the PROCESS macro.

**Results:**

The findings showed that English use on social media was significantly and positively related to interpersonal communication willingness (*r* = 0.283, *p* < 0.01) and directly positively predicted it (*β* = 0.196, *p* < 0.001). Three significant indirect pathways were identified: language confidence (effect size = 0.044, 95% CI [0.011, 0.088]), English cultural identity (effect size = 0.038, 95% CI [0.001, 0.082]), and the chain mediation of language confidence → English cultural identity (effect size = 0.005, 95% CI [0.001, 0.015]).

**Discussion:**

These results indicate that English use on social media is related to college students’ willingness to engage in interpersonal communication via both direct and indirect routes. All identity-related findings in this paper refer specifically to English cultural identity; local, multidimensional, or “dynamic” identities were not empirically measured. Such findings provide a framework for studying the relationship between language use and interpersonal communication within social media contexts and have practical implications for educational and platform design.

## Introduction

Social media is an inseparable thing in life, especially in the era of full-fledged globalization and the rapid development of internet technologies. There are more than 4 billion social media users in the world, and the popularity of social media had a significant impact on social trends and interpersonal communication practices (Hanandini, 2024). College students being a high frequency user group (Alshammari et al., 2023), their social media behavior is directly linked to social relations with others. In the meantime, the widely accepted view on English as a lingua franca is highly widespread within the social media (Sabilla and Kaniadewi, 2025).

But, although the global language used in the context of online communication is English, it is not used in an ideologically neutral way. The fact that the promotion of English as a global resource can mask the power dynamics, linguistic inequalities, and cultural unequal relations inherent in the process of its circulation has long been pointed out by theorists in critical applied linguistics (Phillipson, 1992; Pennycook, 2007; Kubota, 2016). Therefore, when studying the use of English in the social media, it is crucial not only to pay attention to communicative affordances but also to consider the social and ideological environment in the broader context of which learners interact with the language. In this study, we explicitly focus on English cultural identity, defined as identification with English-language cultural symbols, practices, and meanings encountered on social media; we do not measure local or dynamic identities.

Students in non-English-speaking societies such as China, where the English language is a high-status foreign language that is linked to academic success, social status, and internationalization (Dong et al., 2023; Shang, 2024; An et al., 2024) are influenced by their language and cultural ambition to use English in social media. The proficiency in the English language is frequently considered as the linguistic capital, which provides them with enhanced professional and educational opportunities, and this sociopolitical nature of the English language influences the motivation and perception of the English language among the students. Clarifying this context is essential for interpreting what we mean by English use on social media and English cultural identity in this study.

Past studies on the social media and the interpersonal relationships among college students have led to useful results. The impacts of utilizing social media on the social skills of students, the quality of their relationships, and the sense of loneliness have been a subject of numerous studies (Cristea et al., 2024; She et al., 2023). Nevertheless, the linguistic aspect of the use of social media, especially the use of English has not been isolated or intensively discussed by most of these studies. Although certain studies investigated the relationship between language learning and willingness to communicate in second language or foreign learners (Du and Daniel, 2024; Tai, 2024), there remains a gap concerning how English use on social media relates to college students’ willingness to communicate through the mechanisms of language confidence and English cultural identity (MacIntyre et al., 1997).

To address this gap, the present research treats English use on social media as a distinct predictor and examines its effects on students’ interpersonal communication intentions via dual and chained mediation of language confidence and English cultural identity. We also take linguistic ideology as an interpretive lens for understanding why English use on social media may normalize English-linked cultural meanings and thereby relate to stronger English cultural identity (Voci and Karmasin, 2024).

The further exploration of the impact of the English usage on the interpersonal communication intentions of the college students within the social media is of significant value. Theoretically, the study will enrich and complement the frameworks discussing the overlap of language, English cultural identity, and interpersonal communication in the digital space and provide new information on future research. In practice, it can lead college students to make better use of English-language resources in social media so that they can acquire better intercultural communication competencies (Heintz and Scott-Phillips, 2023). In addition, the results can guide the teaching fraternity to formulate instructional programs that may allow students to acquire the communicative and intercultural skills.

**Figure 1:**
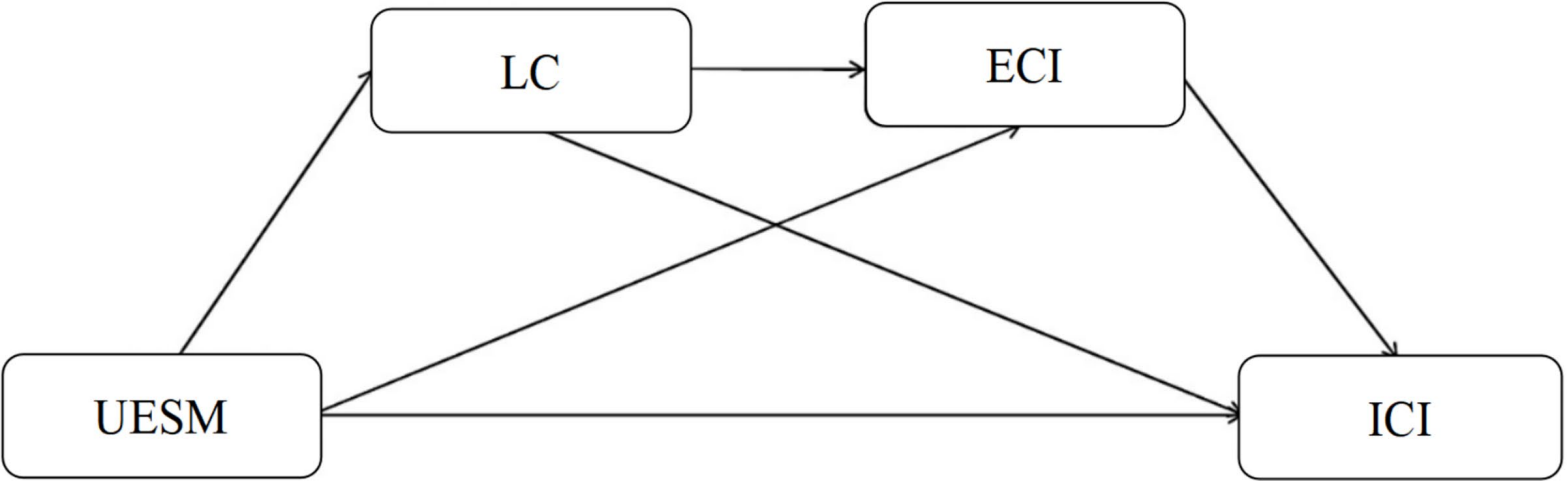
Hypothesis model. UESM, Use of English on social media; ICI, interpersonal communication intentions; LC, language confidence; ECI, English cultural identity.

This research paper assumes that the mediating effect of language confidence and English cultural identity between use of English in social media and readiness of college students to partake in interpersonal communication may be a chain effect. Particularly, the English use in social media boosts the language confidence among the students that is associated with stronger English cultural identity, and finally, English cultural identity relates to the readiness of engaging others. All identity-related statements in this paper refer specifically to English cultural identity; local or multidimensional/dynamic identities are not empirically measured in this study. A detailed analysis of this mediating mechanism can thus clarify how social media English use is associated with interpersonal communication, while keeping the scope of identity strictly limited to English cultural identity (Figure 1).

## Hypotheses

Building on the theoretical analysis presented earlier, this study proposes the following hypotheses:

### Direct effects

*H1*: English use on social media has a significant positive effect on college students’ interpersonal communication intention.

*H2*: English use on social media has a significant positive effect on college students’ language confidence.

*H3*: English use on social media has a significant positive effect on college students’ English cultural identity.

### Mediating effects

*H4*: Language confidence mediates the relationship between English use on social media and interpersonal communication intention.

*H5*: English cultural identity mediates the relationship between English use on social media and interpersonal communication intention.

*H6*: Language confidence and English cultural identity jointly form a chain mediation between English use on social media and interpersonal communication intention.

## Research methodology

### Research participants

This study was conducted using data that was collected in 3 universities in Zhejiang Province, China, which is a non-Anglophone nation. The research participants were chosen as college students since they are the largest participants of the social media, and English learning and use is part of both their studies and their everyday culture, which fit well with the objectives of the study. Convenience sampling technique was used and questionnaires were distributed through the online survey questionstar. A total sample of 412 valid responses were gathered to be analyzed, although the sample includes students of three types of universities (comprehensive and science-oriented ones) all of them were based in Zhejiang Province. Thus, it is possible that the results will not fully generalize to the population of college students in other parts of China, with different linguistic, cultural and educational experiences. Of the participants, 186 were male (45.1%), and 226 were female (54.9%), 101 were first year students (24.5%), 124 were second year students (30.1%), 113 were third year students (27.4%), and 74 were fourth year students (18.0%); The respondents were all familiar with using social media and used their English language features in social networks, being voluntary participants of the survey, which guarantees the representativeness of the data. The research protocol was approved by the Ethics Committee of Zhejiang A&F University (ZAFUAC202550) and adhered to the ethical standards outlined in the 1964 Declaration of Helsinki and its later amendments. All participants signed an informed consent form.

### Research tools

#### Social Media English Usage Scale

The Social Media English Usage Scale was based on the revised version of the Social Media Language Use Questionnaire (SMLUQ), created by Shi et al. (2014), and modified along with the scale Intercultural Communication English Use Scale, suggested by Wang et al. (2020). The instrument assesses via a 5-point Likert scale (1 = never to 5 = very frequently) 10 items that are divided into three dimensions Categorization frequency of social interaction (e.g., I often use English to comment on or like posts on social media) Categorization content production behavior (e.g., I post original status updates or blog posts in English) Categorization level of cross-cultural engagement (e.g., I actively join English-dominated interest communities). Internal consistency of the scale was also great with the Cronbachs alpha of 0.89 in the pilot study. The confirmatory factor analysis showed that the fit in the model was good (CFI = 0.93, RMSEA = 0.06). All the items underwent expert validity tests (Kappa = 0.82) and reverse-scoring items have been incorporated to eliminate the response bias.

#### Willingness to Communicate Scale

In this work, the Willingness to Communicate Scale (WTC) by McCroskey and Richmond (1992) is used as a background with some modifications that Yu (2021) makes to the revised scale adapted to the working conditions of cross-cultural settings. The scale will consist of 15 points, rated on a 5-point Likert scale (1 = very unwilling to 5 = very willing) and it will measure three domains, which include the face-to-face communication (e.g., I am willing to discuss my academic issues with my classmates in person), social media interaction (e.g., I am willing to discuss my academic issues with native English speakers using English-language social media platforms), and cross-cultural contexts (e.g., I am willing to initiate conversations with native English speakers using the English-language social media). The scale has high internal consistency reliability (Cronbachs 87) in the pre-study, and the confirmatory factor analysis has the verified sufficient construct validity (CFI = 0.91, RMSEA = 0.07). The scale was translated into and back-translated to make it linguistically equivalent and then was confirmed using expert reviews (Kappa = 0.85) and cognitive interviews to evaluate the clarity of items.

#### English Language Confidence Scale

Cultural identity is operationalized in this study as English cultural identity, defined as identification with English-language cultural symbols, values, practices, and communicative styles encountered on social media. We do not measure local identities or dynamic identities. The scale was constructed as a 5-point Likert scale (1 = completely disagree to 5 = completely agree) of 6 items, and as suggested by the theoretical framework of language confidence presented by Clément and Kruidenier (1985), and revised through the means of incorporating the Language Confidence Scale (LCS) provided by Dörnyei (2005), the scale targets 3 major dimensions, such as language ability confidence (e.g., I can express my thoughts in English accurately), cross-cultural communication confidence (e.g., I can communicate). The Chinese version of the scale was a validated scale that was adopted in the pre-study. The scale was found to have an overall Cronbachs alpha of 0.88 with the internal consistency coefficients of each dimension ranging between 0.79 and 0.85. A confirmatory factor analysis showed that it fits the model well (CFI = 0.92, RMSEA = 0.06).

#### Cultural Identity Scale

According to the framework of multidimensional cultural identity theory of Phinney (1992), this scale was reworked in parallel with the Digital Cultural Identity Scale (DCIS) designed by Benet-Martínez et al. (2021). The scale is based on the 6-point Likert scale (1 = strongly disagree, 6 = strongly agree) of 6 items representing three main dimensions, such as, value identity (e.g., I identify with the central values of the English culture) and behavioral identity (e.g., I actively engage in the festions of the English culture). The scale in the pre-study has a total Cronbachs 8 coefficient of 0.91, internal consistency coefficients of between 0.83 and 0.88 in each dimension. The model was found to have good fit with the confirmation of confirmatory factor analysis (2/df = 2.36, CFI = 0.94, RMSEA = 0.05). The scale was subjected to two-way translation and back translation in order to establish cross cultural equivalence and later completed the process of content validity using expert review (mean Kappa = 0.87) and focus group interviews.

### Data statistics and analysis

Descriptive statistics, correlation analysis, and common method bias tests were performed using SPSS 26.0. The chained mediation model was validated using the SPSS macro program Process Model 6.

## Results

### Common method bias test

The analysis included 37 items and extracted seven factors. The first factor explained 12.306% of the total variance, which was far below the critical value of 50%, indicating that common method bias was unlikely to have a serious impact on the study results.

### Correlation analysis

The results of the correlation analysis indicate that all study variables are significantly and positively correlated with one another (see Table 1). Specifically, the use of English on social media was positively correlated with interpersonal communication intentions (*r* = 0.283, *p* < 0.01), language confidence (*r* = 0.273, *p* < 0.01), and English cultural identity (*r* = 0.389, *p* < 0.01). Language confidence was positively correlated with both interpersonal communication intentions (*r* = 0.245, *p* < 0.01) and English cultural identity (*r* = 0.268, *p* < 0.01). In addition, English cultural identity was positively correlated with interpersonal communication intentions (*r* = 0.231, *p* < 0.01). Collectively, these findings provide an empirical foundation for the subsequent mediation analysis and suggest patterns of association among the variables, rather than causal relationships.

**Table 1:** Descriptive statistics and correlation analysis of each variable.

Variable	M	SD	UESM	ICI	LC	ECI
UESM	2.894	0.601	1			
ICI	2.977	0.359	0.283**	1		
LC	2.936	0.647	0.273**	0.245**	1	
ECI	4.852	1.281	0.389**	0.231**	0.268**	1

**p < 0.01.

### Chain mediation model testing

Chain mediation model testing was performed using the SPSS macro program Process, and validation was performed using Model 6 to investigate the mediating effect of language confidence and cultural identity. The specific results are as follows:

The results of the structural equation modeling (SEM) analysis (see Tables 2, 3) indicate that the use of English on social media is statistically associated with interpersonal communication intentions through a chained mediation involving language confidence and English cultural identity. Specifically, English use on social media showed significant positive paths to both language confidence (*β* = 0.273, *p* < 0.001) and English cultural identity (*β* = 0.342, *p* < 0.001), while language confidence was positively associated with English cultural identity (*β* = 0.174, *p* < 0.001). When English use on social media, language confidence, and English cultural identity were included in the model simultaneously, all three variables showed significant positive associations with interpersonal communication intentions (*β* = 0.196, *p* < 0.001; *β* = 0.162, *p* < 0.01; *β* = 0.111, *p* < 0.05).

**Table 2:** Testing the chain mediation of English language confidence and cultural identity.

Dependent variable	Independent variable	R	R^2^	F	β	t
ICI	UESM	0.283	0.081	35.752***	0.283	5.979***
LC	UESM	0.273	0.074	32.970***	0.273	5.742***
ECI	UESM	0.424	0.180	44.824***	0.342	7.346***
	LC				0.174	3.743***
ICI	UESM	0.347	0.121	18.664***	0.196	3.815***
	LC				0.162	3.298**
	ECI				0.111	2.164*

*p <0.01, **p < 0.001, ***p < 0.05.

**Table 3:** Bootstrap test of chain mediation effects.

Path	Effect	BootSE	BootLLCI	BootULCI
Total effect	0.283	0.047	0.190	0.376
Direct effect	0.196	0.051	0.095	0.297
Indirect effect	0.087	0.033	0.029	0.157
UESM → LC → ICI	0.044	0.020	0.011	0.088
UESM → ECI → ICI	0.038	0.020	0.001	0.082
UESM → LC → ECI → ICI	0.005	0.004	0.001	0.015

**Figure 2:**
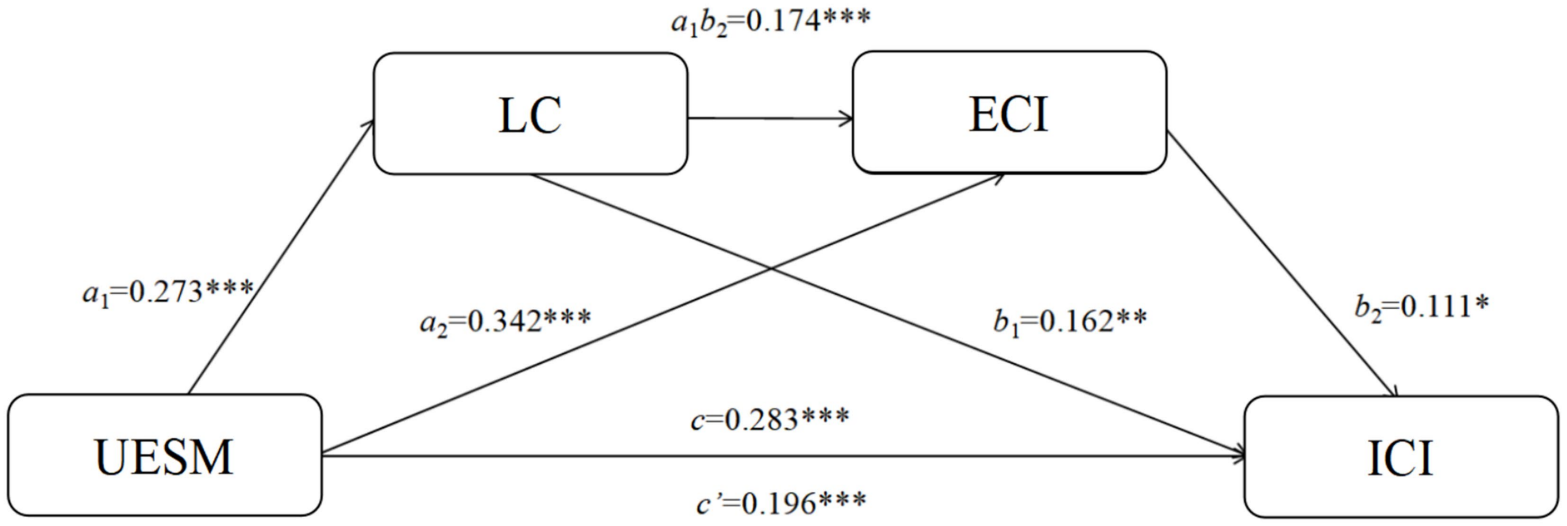
Model validation.

The bootstrap analysis further confirmed these mediating effects (see Table 3; Figure 2). The total indirect effect was 0.087 (95% CI [0.029, 0.157]), accounting for 30.7% of the total effect. Three specific mediating pathways were identified: (1) English use on social media → language confidence → interpersonal communication intentions (effect = 0.044, 95% CI [0.011, 0.088]); (2) English use on social media → English cultural identity → interpersonal communication intentions (effect = 0.038, 95% CI [0.001, 0.082]); and (3) English use on social media → language confidence → English cultural identity → interpersonal communication intentions (effect = 0.005, 95% CI [0.001, 0.015]). All identity-related effects reported here refer specifically to English cultural identity; local or dynamic identities were not measured in this study. Finally, the overall model demonstrated good fit (χ^2^/df = 2.31, CFI = 0.97, TLI = 0.95, RMSEA = 0.056, SRMR = 0.038), providing robust support for the hypothesized chained mediation model.

Although the chained mediation pathway (English use on social media → language confidence → English cultural identity → interpersonal communication intentions) was statistically significant, its effect size was relatively small, explaining only 5.7% of the total indirect effect. This indicates that while both mediators jointly contribute to the relationship between English use on social media and interpersonal communication intention, their combined influence is modest compared with the overall model effect.

## Discussion

This paper evolved a mediating model that included Language Confidence and English cultural identity to investigate the processes by which English on Social Media modulates the Interpersonal Communication Intentions of college students. The findings show that the role played by English on Social Media not only has a direct positive significant role on the Interpersonal Communication Intentions of the students but also there are three important indirect effects, that is, the independent mediation of Language Confidence, the independent mediation of English cultural identity, and sequential mediation of Language Confidence through English cultural identity. For clarity of scope, all identity-related findings in this paper refer specifically to English cultural identity; local or dynamic identities were not empirically measured. In addition, we retain linguistic ideology as an interpretive lens: positioning English as a valued communicative code on social media may normalize English-linked symbols and practices, helping to explain the observed association with English cultural identity and, in turn, interpersonal communication intentions.

The findings can be used to enhance the empirical knowledge of the role of digital language practices in influencing the communicative practices and to contribute to the existing literature on the topic of second language acquisition, intercultural communication, and use of social media. Interpretations and implications throughout this section are therefore confined to English cultural identity as operationalized in our measures. In addition to the theoretical suggestions, the findings can be of practical value to teachers and policy makers indicating that building Language Confidence and English cultural identity as part of learning and development can contribute to the readiness of students to interact in cross-cultural communication in forums both online and off-line.

### The direct effect of social media English use on interpersonal interaction intentions

The results are highly supportive of Hypothesis H1, according to which there is a significant positive correlation between the use of the English language on social media on interpersonal interaction intention of the respondents who are college students. This finding accords with Akpan (2021), who claims that social media has no geographical limits and provides language learners with a wider communication platform. Students who regularly communicate in English via the web regularly find it simpler to widen their existing social networks consequently developing more proactive inclination toward inter-personal communication. In terms of the social capital theory, the language-use social media could be viewed as the process of the gathering of the language social capital. It is the informational resources and interpersonal contacts that are gained in such interactions which gradually enhance the social motivation of the students (Farsi, 2021). As an example, students discussing anything in English in global forums and getting positive feedbacks in other countries, this positive reinforcement makes the students more willing to engage in cross cultural communication.

It is important to note that, the direct effect in this study was less than the total one and therefore, that mediating variables are essential in relationship between social media English use and interpersonal interactions intentions. Simultaneously, existence of a pronounced direct effect makes one think that there is more to be explained. It may be possible that the use of social media in English brings about awareness of global citizenship (Al-Zadjali, 2024), and consequently, encourages the students to interact with other people with different cultures. The second possible mechanism is that the frequent encounters with English language could decrease the levels of social anxiety among students (She et al., 2023), and, therefore, increase their general willingness to communicate. These other processes should be empirically investigated using future studies.

### The mediating role of language confidence

If the use of English on social media significantly and positively predicts language confidence, and language confidence mediates the relationship between English use on social media and college students’ willingness to engage in interpersonal communication, such results would be consistent with the findings of previous studies. Ghafar (2023) and Narzillayevna (2024) both reported that frequent language practice enhances individuals’ confidence, which in turn promotes greater social participation.

From the perspective of second language acquisition theory, social media provides college students with a “low-anxiety” language practice environment (Cadiz-Gabejan, 2021). Unlike the pressure caused by immediate feedback in classroom teaching, online interactions have asynchronous characteristics, such as the ability to delay responses and edit content, which significantly reduce college students’ fear of making mistakes when using English, making them more likely to have successful communication experiences. This positive cycle of “success-confidence-repetition” effectively explains why the use of English on social media can enhance college students’ language confidence. For example, a college student expresses their opinion on a movie in English on social media. Even if there are some grammatical errors, receiving understanding and responses from other users creates a successful experience that boosts their confidence in their English expression abilities, making them more willing to engage in further English communication in the future.

The efficacy of language confidence on interpersonal communication intentions is further confirmed by the receiver of the efficacy transmission mechanism which is the self-efficacy level. When the people feel that they can successfully use language as a means of communication, there is the propensity that they are more likely to start communicational activities and engage people (Dörnyei, 2005). This feeling of communicative self-efficacy gains specific importance in the context of social media. Indicatively, learners who have a greater confidence in language will take the initiative to initiate topic discussions in English-language societies and instigate responsive replies to peers and thus increase their social network as well as increasing their communicative interactions.

The current study indicates the uniqueness of the real-life situation of social media in comparison to Tai (2024) work in which interactions with smart assistants could enhance the willingness of learners to communicate. In particular, the study observed that the so-called genuine social goals (i.e., being liked or finding cross-border friends) are more effective compared to interactions based entirely on tools in enhancing the association between language confidence and readiness to communicate. This can be credited to the fact that genuine social reactions are more effective in citing the persons intrinsic belonging necessity (Rouchy, 2002). When the college students acquire recognition and acceptance of peers through the process of socialization using English, the same feeling of identity encourages them to undertake more and more interpersonal communication.

Also, given that cultural identity is a dynamic and negotiated process, the act of students using English on Social Media may at the same time create and develop one in the context of the changing sense of self in the conditions of multilingualism and multiculturalism. Herein lays the fact that language confidence and cultural identity are inter-related when determining interpersonal communication intentions, and that cultural adaptation, such as exposure to new linguistic practices, affects the motivation of students to communicate across cultural borders.

Consistent with our interpretive focus, we understand these effects in light of linguistic ideology: the framing of English as a prestigious communicative resource online can make English-linked practices more salient and legitimate, which helps explain why greater English use relates to higher language confidence and subsequent outcomes.

### The mediating role of English cultural identity

Hypotheses concerning English cultural identity were supported, indicating that the use of English on social media positively and significantly predicts English cultural identity, and that English cultural identity mediates the relationship between social media use in English and college students’ willingness to engage in interpersonal interactions. This aligns with perspectives that language contact is an important pathway for identity linked to English-language cultural symbols and practices in digital contexts. We emphasize that our conclusions concern English cultural identity as operationalized in the scale; local or dynamic identities were not measured.

The contents of English on the social media does not merely pass the linguistic knowledge but also a goldmine of the Western cultural iconography, i.e., festival rituals and system value. College students communicate in English when socializing, which means that they unconsciously engage in a process of cultural decoding and meaning construction of these cultural symbols (Lestari et al., 2023). As an example, once students engage in the discussion in English speaking communities in regards to Christmas, Thanksgiving and other holidays, they learn about the origin, customs and cultural meaning of such occasions. This can increase their emotional attachment to such cultures which can give them a sense of belonging. Moreover, by initiating to copy the communicative patterns of the English speaking users and attend the cultural holiday celebrations, this can possibly aid in behavior identification. The development of English cultural identity, in turn, makes them more open to socializing with the representatives of the cultural group (Al-Zadjali, 2024), since, in terms of the social-psychological concept, people in general are inclined to have relationships with the groups to which they belong (Karst, 1985). As an example, a college student who shares the culture of the American basketball will more likely discuss the sport with basketball lovers in the United States and share their opinions on the same.

Regarding “conflict effects,” our instrument was not designed to detect tensions with local identity because it measured identification specifically with English-language cultural references; therefore, no inferences are made about local identity or bidimensional identity dynamics.

Accordingly, interpretations should be confined to English cultural identity rather than “one’s own culture” or broader dynamic identity constructs that were not assessed in this study.

### The chain-mediated effect of language confidence and English cultural identity

Assumption H6 was partially supported, with the chain-mediated effect of “social media English usage → language confidence → English cultural identity → willingness to engage in interpersonal communication” being significant, though the effect strength was relatively weak, accounting for only 5.7% of the total indirect effect. This result reveals a dynamic transmission relationship among the variables but also indicates that this pathway occupies a secondary position in the overall influence mechanism. The existence of chain mediation validates the progressive logic of “language - culture - social interaction.” Enhanced language confidence reduces psychological barriers for college students in cross-cultural interactions (Clément and Kruidenier, 1985). When college students are confident in their English communication abilities, they are more willing to actively explore English cultural content, such as reading English news reports, watching English documentaries, and participating in discussions related to English culture. Through these activities, their understanding of English culture gradually deepens, thereby strengthening English cultural identity. In turn, enhanced English cultural identity motivates individuals to practice their cultural understanding through interpersonal interactions (Yu, 2021). For example, a college student who engages in an in-depth and smooth debate in English on a social topic with foreign peers on social media may greatly enhance their language confidence. Subsequently, they may become more proactive in understanding the social and cultural background of that country, gradually forming an identity with that culture in the process. This identity will ultimately motivate them to actively participate in international academic exchange activities and interact with more people from different cultural backgrounds.

The relatively weak path effect may be attributed to several factors. On one hand, the formation of English cultural identity relies more heavily on the depth of exposure to cultural content, such as sustained engagement with English-language influencers who offer critical commentary and cultural perspectives on social and cultural issues, rather than on language confidence alone. Even when a college student demonstrates strong language confidence, if their English usage is confined to surface-level interactions on social media without engaging with the substantive aspects of English language culture, it is unlikely to foster a strong sense of English cultural identity. On the other hand, some students may perceive English as a “neutral tool,” using it exclusively as a medium for information exchange. Increases in language confidence may not necessarily be accompanied by shifts in English cultural identity, which could attenuate the strength of the chain effect. For instance, some students engage in academic discussions in English on social media, prioritizing problem-solving over considering the cultural implications embedded in the language. Consequently, increases in language confidence do not necessarily result in shifts in English cultural identity, thereby limiting its capacity to affect intentions for interpersonal interaction via English cultural identity.

### Limitations and future prospects

Despite the fact that this study uncovered processes by which English use on social media affects college students’ willingness to engage in interpersonal communication, it has several limitations. Regarding methodology, the cross-sectional survey design measures associations among variables but cannot establish causal directionality. For example, it is unclear whether English use on social media enhances language confidence or whether students with higher language confidence are more likely to use English on social media. Future research could employ longitudinal or experimental designs (e.g., interventions manipulating the frequency of English use on social media) to examine causal pathways. Convenience sampling was used in this study. Although the sample included 412 valid responses diversified by gender, grade, and major, it may be biased toward more active social media users, limiting representativeness of the national college student population. Future studies could adopt stratified random sampling to improve generalizability. Importantly, throughout this paper all identity-related analyses and interpretations refer exclusively to English cultural identity; local or multidimensional identities were not empirically measured.

On the measurement side, the cultural identity construct in this study was operationalized solely as English cultural identity and did not include local-identity dimensions, precluding tests of dual-identity interactions (e.g., how local identity and English cultural identity jointly shape interpersonal communication intentions). Accordingly, any mentions of “one’s own culture” or dynamic identity should be read as theoretical background rather than empirical findings in this dataset. Future work should develop a broader, multidimensional index that explicitly measures both local and global identity facets, enabling tests of negotiated/dynamic identity processes. In addition, the study did not include potential moderators such as English proficiency and social anxiety, which may shape the observed relationships. For instance, higher English proficiency could strengthen the mediating role of language confidence by making English communication more fluent and less anxiety-inducing, whereas higher social anxiety might dampen willingness to engage in interpersonal interaction regardless of language confidence.

Moreover, the study did not examine platform-specific differences in English use across social media (e.g., academic vs. entertainment platforms). Distinct communicative purposes, such as knowledge sharing compared with casual interaction, may yield different association patterns among English use, confidence, English cultural identity, and communication intentions. Future studies should compare platform contexts to identify how affordances and norms influence English use and the development of English cultural identity and interpersonal communication intentions, especially when distinguishing professional from informal channels.

Future research can proceed in several directions. First, adopt diary/experience sampling to capture short-term fluctuations in English use and interpersonal communication intentions, allowing tests of within-person dynamics. Second, incorporate moderators such as English proficiency and depth of cultural exposure to examine subgroup differences in mechanisms. Third, broaden contexts by comparing multiple platforms (e.g., Facebook, Twitter/X, academic forums) to clarify situational influences. Fourth, when theoretically warranted, build and measure dual- or multidimensional identity frameworks (including local identity) to test cultural integration pathways, while keeping interpretations distinct from the present study’s scope focused on English cultural identity. Finally, complementary neuroscientific methods (e.g., EEG) could probe neural correlates of language confidence and English cultural identity, enhancing ecological validity. As a cross-sectional limitation note, linguistic ideology has been retained in this manuscript as an interpretive lens; future work may also operationalize and measure it directly to test its moderating or mediating roles.

## Conclusion

This paper discussed how the social media use of English affects the intention of college students to participate in interpersonal communication. Findings show that the use of English through social media is directly and positively related to the willingness to communicate in students, and that indirect effects operate through language confidence and English cultural identity, including a chain pathway from language confidence to English cultural identity. The entire indirect effect explained 30.7 percent of the total effect. These results combine second language learning and English cultural identity hypotheses, explaining a pathway from language practice to individual psychology to social behavior, thus adding to the theoretical background of the interrelationship between language and interpersonal communication in social media contexts.

All identity related conclusions in this study refer specifically to English cultural identity. Local identity, multidimensional identity, and dynamic identity were not empirically measured. Maintaining linguistic ideology as an interpretive lens, we understand the findings in light of how English, as a socially valued communicative code on social media, can shape exposure to English linked cultural meanings that are associated with English cultural identity and with willingness to communicate.

On the practical level, the findings indicate that college students can build language confidence and English cultural identity through high quality English interactions on social media. Universities can integrate structured English social tasks into curricula (for example, online debates, cross campus collaboration, peer feedback conducted in English) and reflective activities that help students connect online English language experiences with cultural references encountered on social media. Social media platforms can add features that support comprehension of English cultural references (for example, contextual annotations and feedback tools), which may facilitate cross cultural communication practice.

Future research should broaden sample coverage and employ longitudinal or experimental designs to examine causal processes. It should also directly measure local identity and multidimensional identity if those constructs are of interest, so that comparisons with English cultural identity can be tested empirically. Platform specific conditions (for example, professional platforms and social platforms) may influence the relationships among English use, language confidence, English cultural identity, and communication intentions, and this warrants systematic comparison. Finally, linguistic ideology could be operationalized and measured in future work to test its moderating or mediating roles, rather than serving only as an interpretive lens.

## Data Availability

The raw data supporting the conclusions of this article will be made available by the authors, without undue reservation.
